# Selective Menin Deletion in the Hippocampal CA1 Region Leads to Disruption of Contextual Memory in the *MEN1* Conditional Knockout Mouse: Behavioral Restoration and Gain of Function following the Reintroduction of *MEN1* Gene

**DOI:** 10.3390/cells11244019

**Published:** 2022-12-12

**Authors:** Anosha Kiran Ulfat, Shadab Batool, Fahad Iqbal, Naweed I. Syed

**Affiliations:** 1 Hotchkiss Brain Institute (HBI), University of Calgary, Calgary, AB T2N 4N1, Canada; 2 Cumming School of Medicine (Cell Biology and Anatomy), University of Calgary, Calgary, AB T2N 4N1, Canada; 3 Alberta Children’s Hospital Research Institute (ACHRI), University of Calgary, Calgary, AB T2N 4N1, Canada

**Keywords:** hippocampus, learning and memory, contextual fear conditioning, *MEN1* rescue, alpha 7 nicotinic receptors, AVV viral transfection, neurons, tamoxifen

## Abstract

Cholinergic neuronal networks in the hippocampus play a key role in the regulation of learning and memory in mammals. Perturbations of these networks, in turn, underlie neurodegenerative diseases. However, the mechanisms remain largely undefined. We have recently demonstrated that an in vitro *MEN1* gene deletion perturbs nicotinic cholinergic plasticity at the hippocampal glutamatergic synapses. Furthermore, *MEN1* neuronal conditional knockout in freely behaving animals has also been shown to result in learning and memory deficits, though the evidence remains equivocal. In this study, using an AVV viral vector transcription approach, we provide direct evidence that *MEN1* gene deletion in the CA1 region of the hippocampus indeed leads to contextual fear conditioning deficits in conditional knockout animals. This loss of function was, however, recovered when the same animals were re-injected to overexpress *MEN1*. This study provides the first direct evidence for the sufficiency and necessity of *MEN1* in fear conditioning, and further endorses the role of menin in the regulation of cholinergic synaptic machinery in the hippocampus. These data underscore the importance of further exploring and revisiting the cholinergic hypothesis that underlies neurodegenerative diseases that affect learning and memory.

## 1. Introduction

Synaptic plasticity forms the basis for learning and memory. This is achieved by regulating neuronal network functions that help an animal adjust to its ever-changing environment [1,2,3,4,5]. For instance, the basal forebrain cholinergic network activity plays an important role in the hippocampal circuits underlying learning and memory, which is comprised of the tri-synaptic pathway of the dentate gyrus (DG), cornu ammonis 3 (CA3) and cornu ammonis 1 (CA1) [6,7]. Cholinergic projections from the medial septal (MS) and diagonal band of Broca (DBB) to the hippocampus also influence the formation of spatial memories [8]. Consistent with the idea of extensive cholinergic involvement in learning and memory are the studies demonstrating that an alteration in hippocampal acetylcholine levels [9] or nicotinic acetylcholine receptors (AChRs) during memory encoding and consolidation affects learning in various animal models [10,11,12]. The role of cholinergic transmission in learning and memory is, therefore, of great significance for long-term memory consolidation and may also underlie many disorders, such as the Alzheimer’s disease (AD) and post-traumatic stress disorder (PTSD) [13]. However, the precise mechanisms regulating cholinergic receptor function in the hippocampus remain poorly defined.

We have recently demonstrated that the multiple endocrine neoplasia type 1 (*MEN1*) gene, which encodes the menin protein [14,15], may regulate the expression of nicotinic acetylcholine receptors (nAChRs) in the hippocampus by specifically targeting these receptors to synaptic sites. Menin, a 610 amino acid sequence, is a 67 kDa nuclear protein with 10 exons [16,17] that serves as a tumor suppressor of the parathyroid glands, pituitary gland, and pancreatic islet cells [18]. While *MEN1* has long been known as a tumor suppressor gene, our novel findings vis-à-vis its involvement in cholinergic synapse formation and synaptic plasticity has since extended its role beyond tumorigenesis [19,20,21,22,23,24].

Menin is involved in the modification of histones, regulation of epigenetic cascades, and is associated with metabolic and autoimmune diseases [25]. The *MEN1* gene has been exceptionally well conserved across evolution, and orthologues are found throughout the animal kingdom [20,26]. Furthermore, *MEN1* has also been shown to be required for cholinergic synaptogenesis and the regulation/targeting of nAChR at the glutamatergic synapses of the hippocampus [20]. However, the precise involvement of menin in the regulation of hippocampal cholinergic receptors during learning and memory remains unknown. Because constitutive *MEN1* knockouts are lethal, an alternative approach was needed to generate a conditional knockout (CKO) model to deduce its precise function in the brain. In this study, we attempted to delete *MEN1* by either using a tamoxifen-inducible model by crossing Camk2-cre/ERT2 mice with *MEN1* floxed mice, or by using an AAV-9 viral vector transduction approach to specifically knockout MEN1 in the hippocampus of the animals. The data presented here underscore the importance of the *MEN1* gene in regulating the expression patterns of nicotinic acetylcholine receptors in the hippocampus, and its important role in fear conditioning learning and memory tests. 

## 2. Methods

### 2.1. Animals

All study protocols involving mice were approved by the Animal Care and Use Committee of the University of Calgary and conducted in accordance with Tri-Council guidelines. All animals were allowed access to standard laboratory chow and water ad libitum and were housed in a 12 hr light/dark cycle under standard laboratory conditions. The *MEN1* floxed mice were obtained from Jackson Labs stock no. 005109, and they had loxP sites flanking exons 3 to 8 of the *MEN1* gene. Mice were homozygous for the allele without any abnormalities. The Camk2a-CreERT2 transgenic mice were obtained from Jackson Labs stock no. 012362. These expressed the tamoxifen-inducible Cre recombinase under the control of the mouse calcium/calmodulin-dependent protein kinase II alpha (Camk2a) promoter region. This was useful for studying gain or loss of function in Camk2a-expressing neurons in cortex, hippocampus, and striatum. The CaMK2a-CreERT2 transgenic mice were bred with *MEN1* floxed mice and CreERT2 fusion gene activity was induced with tamoxifen administration. 

Mouse genotyping was performed at the HBI Molecular Core Facility on ear notch samples using the Kapa Mouse Genotyping kit according to the manufacturer’s instructions. Ear tissue from pups between P21–27 was obtained. Here, P21 was chosen as the age for weaning and tamoxifen administration. This was based on the rationale that the absence of the mother from the cages made them independent juveniles. The experimental and the control groups received intraperitoneal injections of tamoxifen to turn on the activity of Cre recombinase. In the tamoxifen 3-day protocol, a concentration of 10mg/mL was prepared and a dose of 100 μL/mouse was injected for 3 consecutive days. The animals in the control group were also injected with the tamoxifen 3-day protocol. Mice were subjected to molecular experiments after a minimum of 1-week post-final injection so that all traces had been fully excreted (in urine/feces) from the mice [27]. The CKO animals and controls were both subjected to the fear conditioning behavioral paradigm. After the reinjection of *MEN1* rescue virus, the animals and their control counterparts were maintained in a half-way house and monitored regularly for their physical health and subsequently tested again for fear conditioning. The sex distribution was kept constant in each experiment and n = 4 per condition (2 males and 2 females per condition, allowing for a sex-balanced n = 4 per condition). 

### 2.2. Molecular Biology

After 2 weeks of injections, the CKO and control mice were tested to determine if the Cre recombinase induced activity had led to menin deletion. Hippocampus tissue was obtained for western blots to confirm the absence of menin in the CKO mice compared to the controls. Membranes were visualized using a LI-COR Odyssey infra-red imager and analyzed using the gel analysis tool in ImageJ.

### 2.3. Camk2a-Cre/ERT2 Cross with tdTomato, a Mouse Reporter Line

There was a possibility that the mice might not have metabolized tamoxifen or that the Cre expression was leaky, thus, leading to the excision of the *MEN1* gene in both the controls and CKOs. To determine the efficacy of each tamoxifen protocol, a breeding pair between Ai14 mice, homozygous for the Rosa-CAG-LSL-tdTomato-WPRE conditional allele, and the CaMK2a-Cre/ERT2 line was set up to determine the optimal concentration of tamoxifen for inducing Cre activity. The Rosa-td-Tomato mice, homozygous for the Rosa-CAG-LSL-tdTomato-WPRE conditional allele, were obtained from Jackson Labs stock no. 007914 with loxP-flanked STOP cassette to prevent transcription of the red fluorescent protein variant tdTomato. Studies have shown that when bred to mice which expressed Cre recombinase, the STOP cassette is deleted in the Cre-expressing tissue with tdTomato fluorescence [28]. At P21, the offspring from the cross were subjected to a tamoxifen 3-day protocol, where a concentration of 10mg/mL was prepared, and a dose of 100 μL/mouse was injected for 3 consecutive days along with a corn oil 3-day protocol. The animals in the control group were injected with corn oil 100 μL for 3 consecutive days. 2 weeks later, floating brain sections from the animals were live imaged for tdTomato’s expression. An n-value of 10 mice was used with equal sex distribution. 

### 2.4. Virus Transduction of Neuronal Cultures

Ready-to-use AAV9 particles produced from pENN.AAV.hSyn.HI.eGFP-Cre.WPRE.SV40 (#105540) were ordered from Addgene. The AAV has synapsin-driven EGFP-Cre expression, and the packaging plasmid, pENN.AAV.hSyn.HI.eGFP-Cre.WPRE.SV40, encodes the adenoviral helper sequences, AAV rep gene, and AAV9 cap gene. The buffer used for this serotype was PBS + 0.001% Pluronic F-68. The purification method was iodixanol gradient ultracentrifugation. The vector titers were determined by quantitative PCR and the value was 3.1 × 10^13^ GC/mL. It was stored at −80 °C and thawed before use on ice. Controls received AAV9 pENN.AAV.hSyn.HI.eGFP.WPRE.SV40 for control GFP expression with a titer of 2.8 × 10^13^ GC/mL, and the in vivo CKO received the Cre AAV9 virus. 

Embryos were dissected from *MEN1* floxed pregnant females on E18 for hippocampal neuronal cultures. The pregnant mice were anesthetized with isoflurane and then sacrificed by decapitation. The hippocampi from E18 embryos were dissected in solution (1 × HBSS containing 10 mM HEPES; 310 mOsm, pH 7.2), and then treated with papain (50 U/mL), 150 mM CaCl2, 100 µM L-cysteine, and 500 µM EDTA in neurobasal medium (NBM) for 20 m at 37 °C and 5% CO_2_ (in an incubator). Then, NBM supplemented with 4% FBS, 2% B27, 1% penicillin-streptomycin, and 1% L-glutamine (GIBCO) was used to wash the tissue 3 times. For trituration, fire-polished glass Pasteur pipettes were used and then plated on glass coverslips. These had been previously washed with nitric acid and coated with poly-D-lysine (30 µg/mL) and laminin (2 µg/mL). The culture media was changed to NBM supplemented with 2% B27, 1% penicillin-streptomycin, and 1% L-glutamine the very next day, and the samples were divided into 2 groups. The controls were supplemented with the media, and the experimental groups was transduced with the AAV9 Cre virus. The cultures were maintained at 37 °C with 5% CO_2_, and the media was changed every 3 to 4 days. 

### 2.5. Immunocytochemistry and Microscopy

Hippocampal neuronal cultures from the control and the experimental groups were fixed on day in vitro (DIV) 21 with 4% paraformaldehyde (Sigma-Aldrich, St. Louis, MI, USA) in 1 × PBS for 30 min. The cells were permeabilized for 1 h with incubation medium (IM) containing 0.5% Triton and 10% goat serum in 1 × PBS. Primary antibody, menin C-terminal epitope [Bethyl Laboratories, A300-105, Montgomery, TX, USA], was used at 1:500 in IM for 1 h. Secondary antibodies, namely Alexa Fluor 488, 568, or 680 (Invitrogen, Vancouver, BC, Canada) were used at 1:100 in IM for 1 h followed by three 15 m washes in 1 × PBS. Cells were mounted using ProLong Gold antifade reagent with DAPI (Invitrogen, BC, Canada). A VS120 microscope at 40X magnification was used for imaging. Fluorophores were excited with 488, 568, and 680 lasers. Imaging parameters, including field of view size, laser intensity, exposure, and channel gain, were kept consistent throughout. 

### 2.6. In Vivo Virus Transduction in CA1 Hippocampus of MEN1 Floxed Mice

The *MEN1* floxed mice were injected with the adeno-associated virus (AAV) under the synapsin promoter for neuron-specific *MEN1* deletion. The hippocampal CA1 neurons and a control AAV were used to express GFP under the synapsin promoter bilaterally into the dorsal hippocampi (2 mm behind bregma, 2 mm lateral, and 1.6 mm below the dural surface for CA1). The injections were performed with glass micropipettes with the tip diameter ranging between 10 to 20 μm and injected slowly with a pressure-injection system. The recovery time post-injection was 4 weeks. To validate this model, immunostaining was performed, where the brains of the controls and CKOs were fixed with 4% paraformaldehyde (PFA) overnight, transferred to 2% sucrose, and then snap-frozen using dry ice and OCT. Brain slices were prepared using cryostat with thickness of 16 μm. The sections were tagged with C-menin, as previously described, to confirm menin deletion. 

### 2.7. Behavioral Paradigm for Contextual Fear Conditioning

The behavioral tests were adapted from the Cumming School of Medicine Optogenetics facility at the University of Calgary. Mice reach sexual maturity between six to eight weeks and, based on the timing of stereotactic injections and recovery time, the time frame for behavior testing was deemed to be two months [29]. The protocol for contextual fear conditioning was two days long, and this test is used to assess hippocampal-dependent learning and memory [30]. On day one, mice were placed in the conditioning chamber, habituated to their surroundings for 2 min, and then were given three consecutive shocks (1 s, 0.5 mA). This was recorded with a camera mounted inside the chamber. On day two, the procedure and context remained the same to test conditioned fear of the shock, but no shocks were administered. Using the ANY-maze software (Stoelting Co., Wood Dale, IL, USA), freezing (defined by the complete absence of motion), as a measure of fear in the rodent model was scored as percentage of time spent frozen and analyzed with GraphPad Prism 8 (Dotmatics). 

### 2.8. AAV Production for MEN1 Rescue Virus

The mouse *MEN1* cDNA rom Horizon Discovery (NCBI accession NM_001168490.1) was PCR-amplified and inserted in place of Cre recombinase at the BspEI and HindIII restriction sites in pENN-AAV-hSyn-HI-eGFP-cre (Addgene plasmid, Teddington, UK #105540) using the NEBuilder HiFi DNA assembly kit, and the pAAV-hSyn-HI-eGFP-MEN1 was generated. The AAV viral vectors, which had the AAV9 capsid, were generated based on methods previously described [31]. The 293FT cells (Thermofisher, Waltham, MA, USA) were co-transfected with 129 µg pHELPER (Agilent, CA, USA ), 238 µg pAAV 2/9n rep-cap plasmid (Addgene plasmid #112865) and pAAV.hSyn-HI-eGFP-MEN1 with the PEIpro transfection reagent. The AAVs were precipitated using 40%PEG/2.5 M NaCl, and the pooled cells were harvested after 5 days in 500 mM NaCl, 40 mM Tris Base, and 10 mM MgCl2 buffer. The lysate was incubated with 100 U/mL salt-active nucleases (Arcticzymes, Tromsø. Norway) at 37 °C for 1 h and then centrifuged at 2000× *g* for 15 min. Using the iodixanol step gradient containing 15, 25, 40, and 60% iodixanol in OptiSeal tubes followed by centrifugation at 350,000× *g*, the AAV was purified from the lysate. Then, using the 10 cc syringe and 16-gauge needle, the AAVs were harvested in 1XPBS containing 0.001% Pluronic F68 (Gibco, Grand Island, NE, USA) and filtered. A qPCR adeno-associated virus titration kit was used for calculating the titer (Expedeon, Heidelberg, Germany).

### 2.9. In Vitro MEN1 Virus Transduction for Testing MEN1 Rescue Virus Efficacy

Embryos were dissected from *MEN1* floxed pregnant females on E18 for hippocampal neuronal cultures. The pregnant mice were sacrificed by decapitation after being anesthetized with isoflurane. The hippocampi from E18 embryos were dissected in solution (1 × HBSS containing 10 mM HEPES; 310 mOsm, pH 7.2), and then treated with papain (50 U/mL), 150 mM CaCl2, 100 µM L-cysteine, and 500 µM EDTA in neurobasal medium (NBM) for 20 m at 37 °C and 5% CO_2_ (incubator). Then, NBM supplemented with 4% FBS, 2% B27, 1% penicillin-streptomycin, and 1% L-glutamine (GIBCO) was used to wash the tissue 3 times. For trituration, fire-polished glass Pasteur pipettes were used and then plated on the glass coverslips. These had been previously washed with nitric acid and coated with poly-D-lysine (30 µg/mL) and laminin (2 µg/mL). The culture media was changed to NBM supplemented with 2% B27, 1% penicillin-streptomycin, and 1% L-glutamine the very next day, and the samples were divided into 2 groups. The controls were supplemented with the media, and the experimental groups were transduced with the *MEN1* rescue virus. The cultures were maintained at 37 °C with 5% CO_2_, and the media was changed every 3 to 4 days.

Hippocampal neuronal cultures from the control and the experimental groups were fixed on DIV21 with 4% paraformaldehyde (Sigma-Aldrich, St. Louis, MI, USA) in 1 × PBS for 30 min. The cells were permeabilized for 1 h with incubation medium (IM) containing 0.5% Triton and 10% goat serum in 1 × PBS. Primary antibody, menin C-terminal epitope (Bethyl Laboratories, A300-105, Montgomery, TX, USA), was used at 1:500 in IM for 1 h. Secondary antibodies, namely Alexa Fluor 488, 568, or 680 (Invitrogen, Vancouver, BC, Canada) were used at 1:100 in IM for 1 h followed by three 15 m washes in 1 × PBS. Cells were mounted using ProLong Gold antifade reagent with DAPI (Invitrogen, BC, Canada). A VS120 microscope at the 40X magnification was used for imaging. Fluorophores were excited with 488, 568, and 680 lasers. Imaging parameters, including field of view size, laser intensity, and channel gain, were kept consistent throughout. 

### 2.10. Reinjection of MEN1 Rescue Virus into the CKO Animals

The CKO mice underwent stereotaxic injections with *MEN1* AAV rescue virus (pAAV-hSyn-HI-eGFP-MEN1), and the controls were injected with PBS bilaterally into the dorsal hippocampi (2 mm behind bregma, 2 mm lateral, and 1.6 mm below the dural surface for CA1). The injections were performed with glass micropipettes with the tip diameter ranging between 10 to 20 μm, and injected slowly with a pressure-injection system. The recovery time post-injection was 4 weeks before the animals were tested again for contextual fear and conditioning task, as mentioned above. 

## 3. Experimental Design and Statistical Analysis

For more robust reproducibility, all of the data were derived from ≥3 independent experiments. Image J 1.53t (NIH) software was used for image processing, calculating the fluorescence intensity, and processing the blot. Quantification tools were kept constant throughout the data acquisition and analysis. Blind analysis was used to reduce the bias by conducting the analysis on acquisition file number. Statistical analyses were performed using the GraphPad Prism version 8.3.1 GraphPad software. Differences in fluorescence intensity for ICC, WB, and the freezing score for the behavioral assessment of hippocampal-dependent fear conditioning were assessed with the Mann–Whitney U test to compare the differences between the groups. `

## 4. Results

### 4.1. Crossing Tamoxifen Inducible Camk2a-cre/ERT2 and MEN1 Floxed Mice Failed to Delete Menin in the CKO Model as Compared to the Control Animals

**Figure 1:**
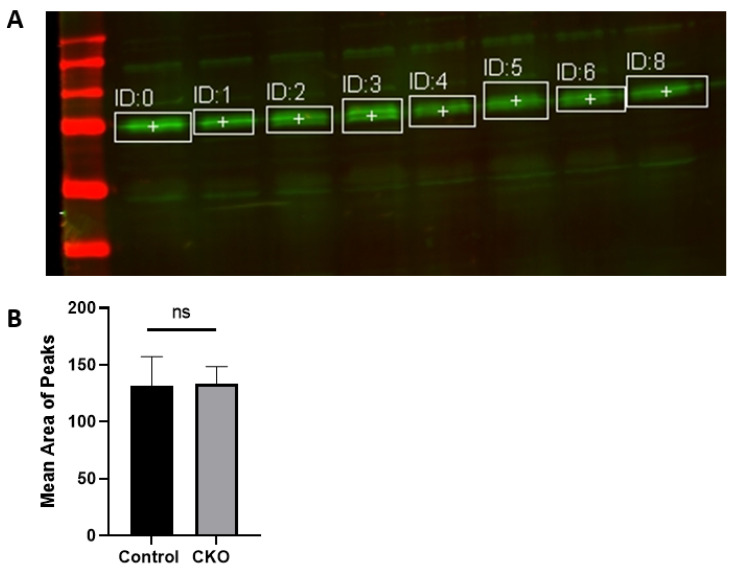
Validation of the CKO model by crossing tamoxifen-inducible CamK2a Cre-ERT2 mice with *MEN1* floxed mice. (**A**) Validation was achieved through WB for comparing menin protein in the CKOs and control animals. The WB analysis of C-menin expression in the hippocampus at P54 with age matched mice. The C-menin expression for the controls is marked in the lanes ID:0, ID:1, ID:2, and ID:3. The C-menin expression for the menin CKO is marked in the lanes ID:4, ID:5, ID:6 and ID:8. (**B**) Summary data, showing no difference in the mean area of the peaks between the controls and the experimental groups. Data represent the mean ratio ± SEM. n = 4. Statistical significance (Mann–Whitney U test) ns *p* > 0.05.

We first sought to determine whether a tamoxifen-inducible Camk2a-cre/ERT2 approach could selectively delete MEN1 in the hippocampal neurons. The mice were injected with tamoxifen, and we next validated the efficacy of the model by determining if any difference could be found in menin expression levels between the CKO and control animals. To confirm the deletion of the *MEN1* gene and menin protein in these animals, we performed protein extraction from the hippocampi and performed western blot (WB) analysis. Based on the mean area of peaks shown in the WB and their relative comparison, no significant difference was found (Figure 1). Thus, menin deletion in the CKO model compared to the controls could not be confirmed. These results were unexpected, and contrary to what we had anticipated for this experiment. 

### 4.2. Cre Recombinase in Camk2-cre/ERT2 Mice Crossed with the tdTomato Line Was Expressed Both in Controls and Tamoxifen Injected Mice 

To further confirm the above negative results, we next sought to determine the expression patterns of Cre recombinase in the Camk2a CreERT2 line. One potential possibility could have been either a leaky Cre expression or no Cre expression in these animals due to insufficient administration of tamoxifen. To further assess this possibility, we tested the tamoxifen protocol by crossing the Camk2 Cre-ERT2 line with a reporter line to determine the activity patterns of Cre in the mice injected with tamoxifen and vehicle (corn oil). 

The Camk2Cre-Ert2 mice were crossed with the tdTomato line to determine the efficacy of the tamoxifen protocol. A breeding pair between Ai14 mice, homozygous for the Rosa-CAG-LSL-tdTomato-WPRE conditional allele, and the CaMK2a-Cre/ERT2 line was, therefore, set to validate this model further. A crucial step before moving on to the next was genotyping the animals. Based on the Jax protocols, we saw that the molecular weight for the Cre transgene, positive control, tdTomato mutant, tdTomato heterozygote, and tdTomato and wildtype were in the range of the genotyping results shown by the JAX lab. 

We anticipated that the animals which had received tamoxifen would have Cre expression, whereas those injected with corn oil would be devoid. In the Ai14 mice, a Cre reporter strain expressed tdTomato fluorescence following Cre-mediated recombination. On the contrary, both the controls and the tamoxifen group showed tdTomato fluorescence, suggesting that the Cre was activated (Supplementary Figure S1). Taken together, although the above results were disappointing, they were an essential step towards testing the efficacy and efficiency of the knockout regime. Since the above approach was not viable, a switch was made to AAV9 serotype for virus transduction, as described below for both in vitro and in vivo. 

### 4.3. In Vitro Virus Transduction Led to Menin Deletion 

**Figure 2:**
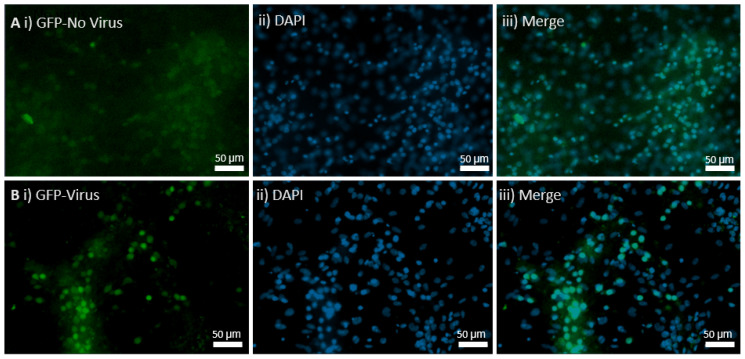
The transduction of hippocampal neuronal cultures from the *MEN1* floxed mice with the virus, pNN.AAV.hSyn.HI.eGFP-Cre.WPRE.SV40. (**A**) Controls with no virus; (**i**) GFP signal in the absence of virus; (**ii**) DAPI for nuclear staining; (**iii**) merge with GFP and DAPI; (**B**) CKO with virus transduction by Cre AAV9; (**i**) GFP signal indicating virus transduction in the preparation with the Cre AAV9; (**ii**) DAPI for nuclear staining; (**iii**) merge with GFP and DAPI showing overlap in the neurons expressing GFP and DAPI.

To test the efficacy and efficiency of AAV9 approach, which has been used effectively in other models to knockout the expression of several genes [32,33], we first validated its utility by testing it on the cultured neurons. Based on the results from the cross between the tamoxifen-inducible approach between the *MEN1* floxed mice and Camk2a Cre-ERT2 mice, our goal was to first determine the efficacy of the virus by simply testing its transduction potential in the hippocampal culture and then observing the GFP expression in the neurons. The AAV9 Cre recombinase viral construct was tagged with GFP construct, and the GFP signaling was used as an indicator of whether the cells were transduced or not. As shown in hippocampal neuronal culture (Figure 2), GFP was tagged in the preparation where the AAV9 Cre was transduced. The virus transduction in the hippocampal neuronal cultures from the *MEN1* floxed mice DIV 21 was compared to that of the controls, which did not receive the AAV9 virus. The transduction of the virus was confirmed by the expression of GFP-expressing cells.

**Figure 3:**
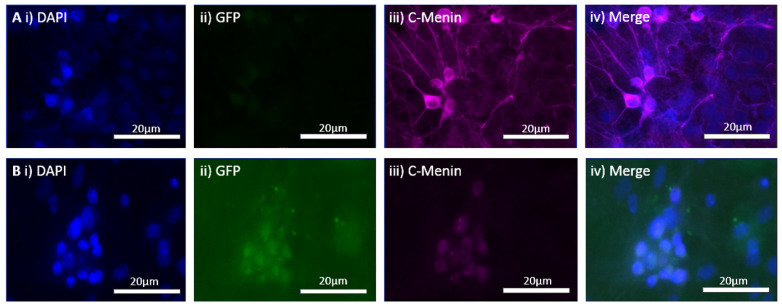

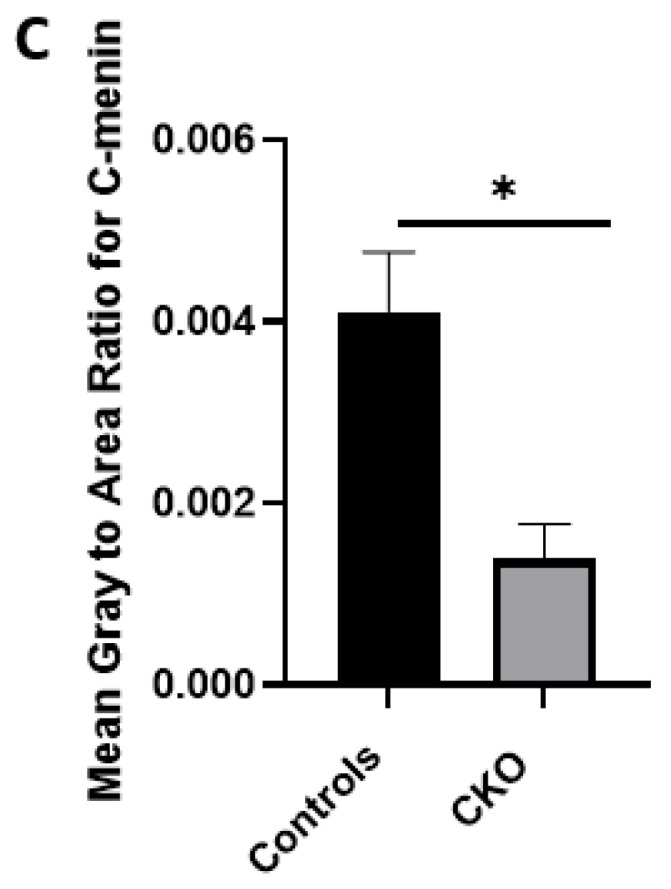
In vitro virus transduction of the hippocampal neurons from *MEN1* floxed mice in control and CKO groups. (**A**) Controls with no virus; (**i**) the expression of DAP for nuclear staining; (**ii**) GFP signal in the absence of virus; (**iii**) C-menin showing axoplasmic expression; (**iv**) merge in controls for GFP and DAPI; (**B**) CKOs with virus; (**i**) DAPI for nuclear staining; (**ii**) GFP signal indicating virus transduction in the preparation with the Cre AAV9; (**iii**) C-menin signal weaker compared to the controls; (**iv**) merge in the CKO for GFP and DAPI; (**C**) summary data, showing the reduction in menin signals as measured by mean gray-to-area ratio in the CKO compared to the controls. Data represent the mean ratio ± SEM. n = 4. Statistical significance (Mann–Whitney U test), * *p* < 0.05.

Having confirmed the expression of GFP with virus transduction, we next sought to determine if any alteration in the expression patterns of menin could be observed in the CKO and control preparations. This was followed by immunostaining both the controls and CKO to determine if the menin expression changed after *MEN1* gene deletion. The mean gray-to-area ratio of the controls was 0.004112, and the mean gray-to-area ratio of the CKO was 0.001380 (Figure 3). The C-menin expression was significantly reduced in the CKO, and the neurons exhibiting C-menin showed reduced dendritic branching in the CKO. 

### 4.4. In Vivo Viral Transduction Led to Menin Deletion in Freely Behaving Mice

The above experiments validated the utility of *MEN1* deletion in live cultures. We next sought to determine if this approach could also be employed to knockout *MEN1* expression in freely behaving animals. The *MEN1* deletion in the hippocampus of the floxed *MEN1* mice was induced via the Cre AAV9 with neuron-specific synapsin promoter. The controls were injected with GFP AAV9 with neuron-specific synapsin promoter. 

**Figure 4:**
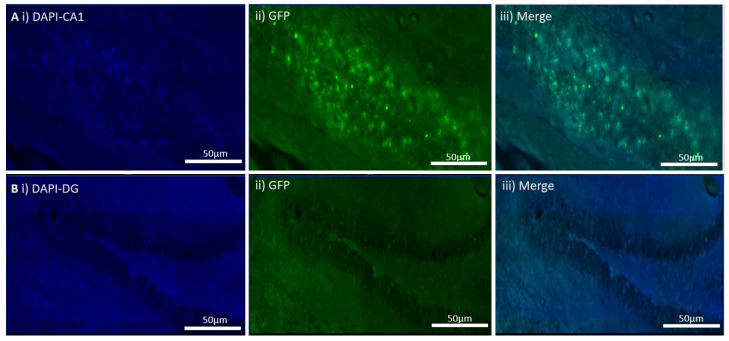
In vivo virus transduction of the hippocampal CA1 from *MEN1* floxed mice in CA1 region of hippocampus and dentate gyrus (DG). (**A**) Expression of GFP in CA1 in the CKO; (**i**) the expression of DAPI for nuclear staining in CA1; (**ii**) the expression of GFP as the marker of virus transduction in the neurons transduced by the AAV9 under the action of synapsin promoter in CA1; (**iii**) merge in controls with control virus AAV9 GFP and overlap between DAPI and GFP; (**B**) non-GFP expressing region in dentate gyrus (DG); (**i**) the expression of DAPI for nuclear staining in DG; (**ii**) GFP signal in the non-transduced region of hippocampus; (**iii**) merge for showing the overlap between DAPI and GFP.

**Figure 5:**
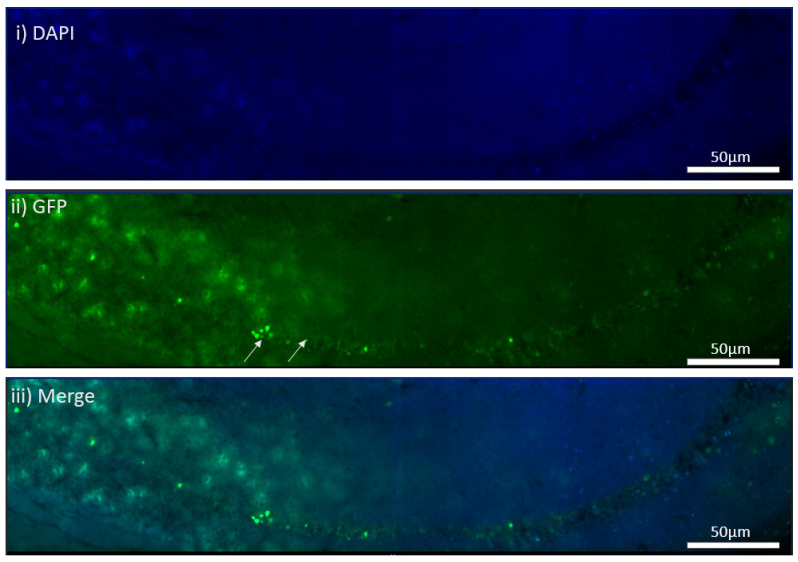
In vivo virus transduction of the hippocampal CA1 neurons in *MEN1* floxed mice. (**i**) DAPI for nuclear staining; (**ii**) GFP expression in the CA1 neurons and the ectopic expression in the vicinity of the CA1 region as pointed by the arrows; (**iii**) merge showing the overlap between DAPI and GFP.

**Figure 6:**
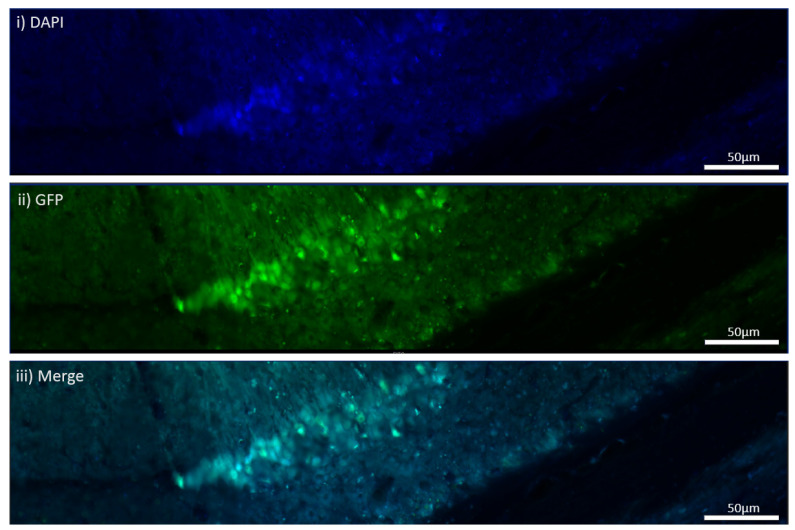
Ectopic expression in the hippocampus of the mice injected with AAV9 under the function of synapsin promoter and GFP as the marker for the virus transduction. (**i**) The expression of DAPI for nuclear staining; (**ii**) GFP as the marker of virus transduction in the neurons transduced by the control AAV9 under the action of synapsin promoter; (**iii**) merge in controls showing overlap between DAPI and GFP.

Four weeks after the Cre AAV9 injections, brain sections were imaged to determine whether GFP expression had occurred or not. The CKO expressed GFP in the CA1 region, whereas the dentate gyrus was devoid of the GFP signal (Figure 4). There was also ectopic expression and, thus, WB and the ICC quantification were conducted from the entire hippocampus and not just the CA1 area (Figure 5). The controls also expressed GFP in the CA1 region of the hippocampus (Figure 6). 

**Figure 7:**
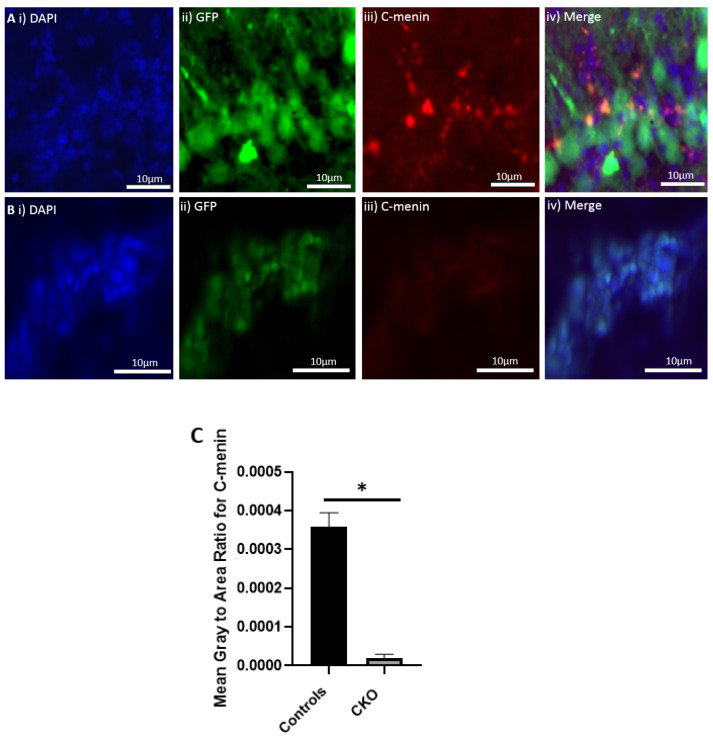
Reduced menin expression in the hippocampal sections of the CKO animals. (**A**) Hippocampal section of the control mice injected with the control virus; (**i**) DAPI for nuclear staining; (**ii**) GFP-expressing neurons after the transduction of the control virus (**iii**). C-menin’s expression in the neurons (**iv**). Merge for showing the overlap between GFP-expressing neurons, DAPI, and C-menin; (**B**) hippocampal section of the CKO mice injected with the Cre-expressing virus; (**i**) DAPI for nuclear staining; (**ii**) GFP-expressing neurons after the transduction of the Cre recombinase-inducing virus; (**iii**) C-menin’s weak signal in the neurons; (**iv**) merge for showing the overlap between GFP expression, DAPI, and C-menin; (**C**) summary data, showing the reduction in menin’s signal as measured by mean gray-to-area ratio in the CKO compared to the controls. Data represent the mean ratio ± SEM. n = 4. Statistical significance (Mann–Whitney U test), * *p* < 0.05.

Once the virus transduction was confirmed in the controls and the CKOS, the next step was to determine if menin was deleted in the CKO model. Using an immunostaining approach, we confirmed that menin was indeed deleted from the AAV9 Cre-expressing neurons. The mean gray-to-area ratio of the controls was 0.0003575 and the mean gray-to-area ratio of the CKO was 0.00001988 (Figure 7). The C-menin expression was significantly reduced in the CKO.

### 4.5. AVV9 Viral Vector Mediated CKO of MEN1 Gene in the CA1 Hippocampal Region Perturbs Fear Conditioning Memory

To test the hypothesis that hippocampal *MEN1* CKO renders fear conditioning, learning, and memory compromised, we injected the mice with AAV9 to conditionally knock out the expression of *MEN1*, leading to the deletion of its encoded menin protein. The control animals were injected with AAV9-synapsin-GFP for the expression of GFP under the synapsin promoter, whereas the CKO animals received AAV9-synapsin-Cre-synapsin-GFP for the expression of Cre recombinase under the synapsin promoter. Specifically, the neuron-specific CKO model of menin was created for the purpose of testing its role in hippocampus-dependent learning and memory. The CKO and control animals were tested for their hippocampal-dependent learning and memory of fear conditioning in a neutral context. Animals of both sexes between the ages of 8 to 10 weeks were used in all experiments. 

**Figure 8:**
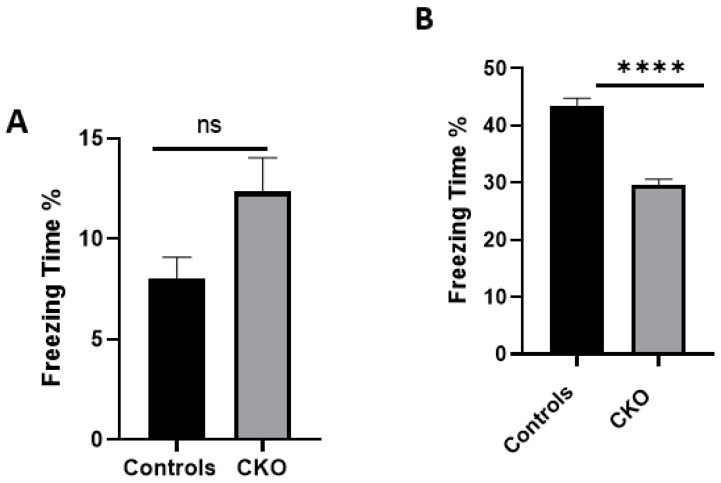
Contextual fear conditioning deficits in the CKO mice. (**A**) For the contextual fear conditioning test, mice were trained and analyzed for freeze response. The scores of the freezing time (%) for the control mice and the CKO on day one show little difference; (**B**) CKO animals had a lower freezing time % score on day two compared to the controls. Freezing response is the percentage of time spent freezing from the total time duration. Data represent the mean ± SEM. n = 16. Statistical significance (Mann–Whitney U test), **** *p* < 0.0001, ns *p* > 0.05.

The health of the animals and their locomotor movements, including feeding patterns, were continuously monitored for 4 weeks post-stereotaxic injections. We anticipated that the viral transduction would have occurred after these 4 weeks, as has been shown by previous studies [34]. The animals were tested over a two-day protocol where, on day one, foot shocks were administered and, on day two, their behavior was scored as % time freezing using the ANY-maze software. The % time freezing is a measure of stress/fear in the animals, and the percentages were calculated based on time (seconds) spent in the freezing state. On day one, the freezing scores as % of the control animals compared to the CKO showed a little difference. The mean % time freezing in CKO was 12.34%, and in controls it was 8.044%; however, the difference was non-significant (Figure 8). We found that on day two, the control animals’ % time freezing score was significantly higher compared to the CKOs. The mean for the CKO was 29.62% and for the controls it was 43.53%. This suggested that compared to the controls, the CKO mice were not able to retain the fear conditioning memory from day one when subjected to the same context on day two. This higher score in the controls compared to the CKO demonstrated that the CKO animals failed to retain the contextual fear conditioning, and that their hippocampal-dependent learning and memory was significantly affected due to the deletion of *MEN1* in the CA1 hippocampus. 

**Figure 9:**
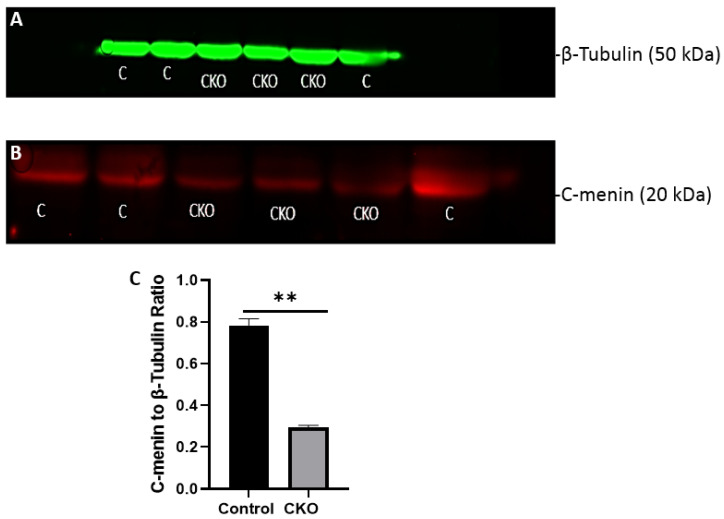
Reduced C-menin expression in *MEN1* CKO post-behavior testing. (**A**) WB of the mouse hippocampal protein samples with β-Tubulin antibody (loading control) showing the expression of β-Tubulin in controls, and CKO showing relatively similar fluorescence across the controls and CKO at the expected molecular weight of 50 kDA; (**B**) WB of the mouse hippocampal protein samples with menin C-terminal epitope antibody for the detection of the C-terminal fragment in the controls (**C**) and the conditional knockout (CKO) animals at the expected molecular weight of 20 kDA, and the bands from the control animals have higher fluorescence compared to the CKOs; (**C**) summary data, showing the comparison of menin-to-β-Tubulin ratio in controls and the CKOs, with a reduction in the C-menin-to-β-Tubulin ratio in the CKO. Data represent the mean ratio ±SEM. n = 4. Statistical significance (Mann–Whitney U test), ** *p* < 0.05.

To validate the specificity of the CKO and to corroborate the behavioral findings with the relative expression of menin protein, we sacrificed the CKO and control animals after the behavior testing was completed and sought to determine if menin was indeed downregulated. To test this, we sought to determine the relative protein expression in both the experimental and the control animals by using western blots (WB). In the CKO animals, the mean ratio of menin to β-tubulin was 0.2928, and in the controls it was 0.7834, indicating significant downregulation of the menin-to-β-tubulin ratio in the CKO compared to the controls (Figure 9). However, since menin is not only found in neurons but also in astrocytes and microglia, there was some expression in the CKO as well [22,35].

### 4.6. MEN1 Rescue Virus Efficacy Was Confirmed in the In Vitro Hippocampal Cultures

**Figure 10:**
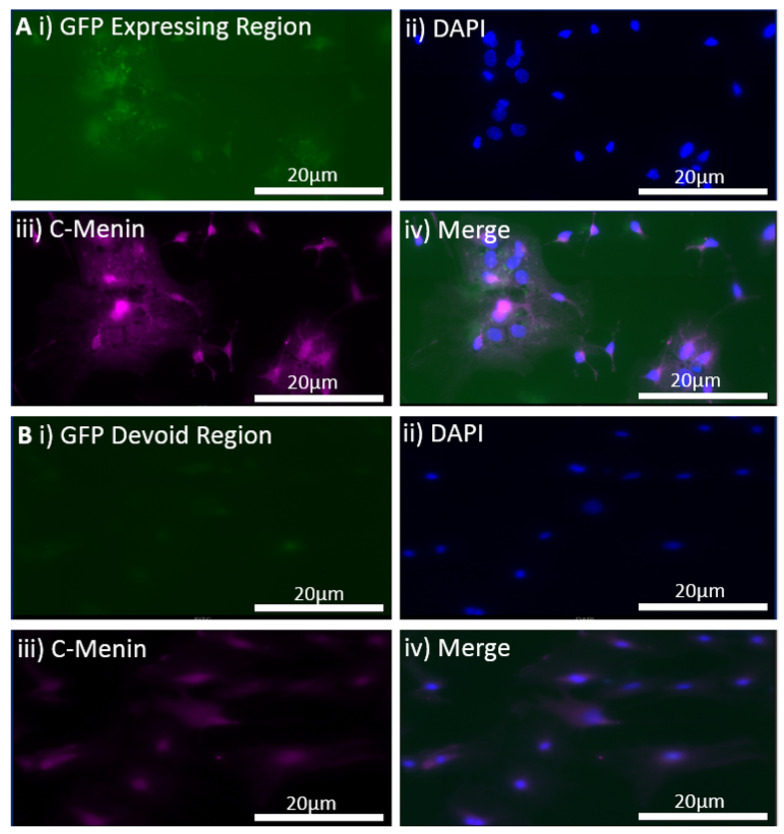

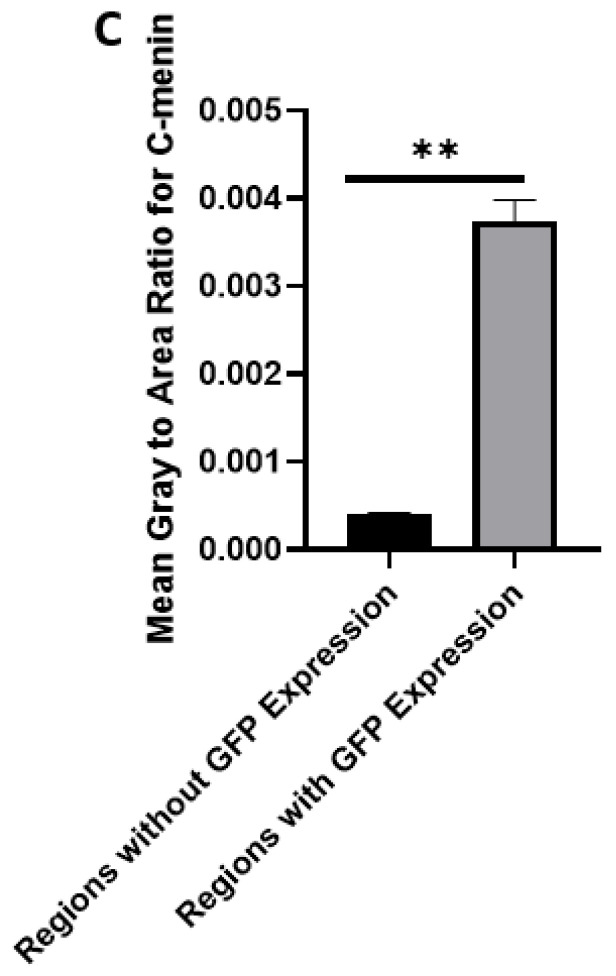
In vitro expression of *MEN1* rescue virus, pAAV-hSyn-HI-eGFP-MEN1, in the hippocampal cultured neurons. (**A**) The GFP-expressing neurons after the transduction of the *MEN1* rescue virus; (**i**) the GFP-expressing neurons; (**ii**) DAPI for nuclear staining; (**iii**) C-menin expression in the neurons; (**iv**) merge for showing the overlap between GFP-expressing neurons, DAPI, and C-menin; (**B**) the non-GFP expressing regions (controls); (**i**) the non-GFP-expressing neurons; (**ii**) DAPI for nuclear staining; (**iii**) C-menin expression in the neurons; (**iv**) merge for showing the overlap between GFP expression, DAPI, and C-menin; (**C**) summary data, showing the mean gray-to-area ratio for Cy5 channel for C-menin in GFP expressing and non-GFP expressing regions, indicating an increase in C-menin expression in GFP-expressing neurons. Data represent the mean ± SEM, n = 4, Statistical significance (Mann–Whitney U test), ** *p* < 0.05.

To test the hypothesis that *MEN1* CKO-induced behavioral deficit could be rescued by the reintroduction of *MEN1* in the intact animals, we first sought to test this possibility in cell culture. We sought to determine whether the transduction of hippocampal neuronal culture with *MEN1* rescue virus results in the overexpression of *MEN1* and its encoded protein menin. The rescue of the *MEN1* gene was verified with the inclusion of a reporter marker, GFP. The technique of the reintroduction was based on the insertion of mouse *MEN1* cDNA obtained from mammalian gene collection in place of Cre recombinase, generating pAAV-hSyn-HI-eGFP-MEN1. This technique simply overexpressed the *MEN1* gene leading to an upregulation of menin protein in the neurons with the synapsin promotor. The GFP-expressing neurons exhibited an increase in the C-menin expression as deduced from immunocytochemistry data when compared to the non-expressing regions. The regions with GFP expression indicative of transduction via the *MEN1* rescue had a mean gray-to-area ratio of 0.003737, and the control regions without GFP expression had a mean gray-to-area ratio of 0.0004021 (Figure 10). Having tested the efficacy of the *MEN1* rescue virus leading to menin overexpression, we next injected the CKO animals with the *MEN1* rescue virus and retested the mice for contextual fear and conditioning. 

### 4.7. MEN1 Reintroduction into CKO Animals Recovers the Contextual Fear Conditioning Behavioral Deficit

**Figure 11:**
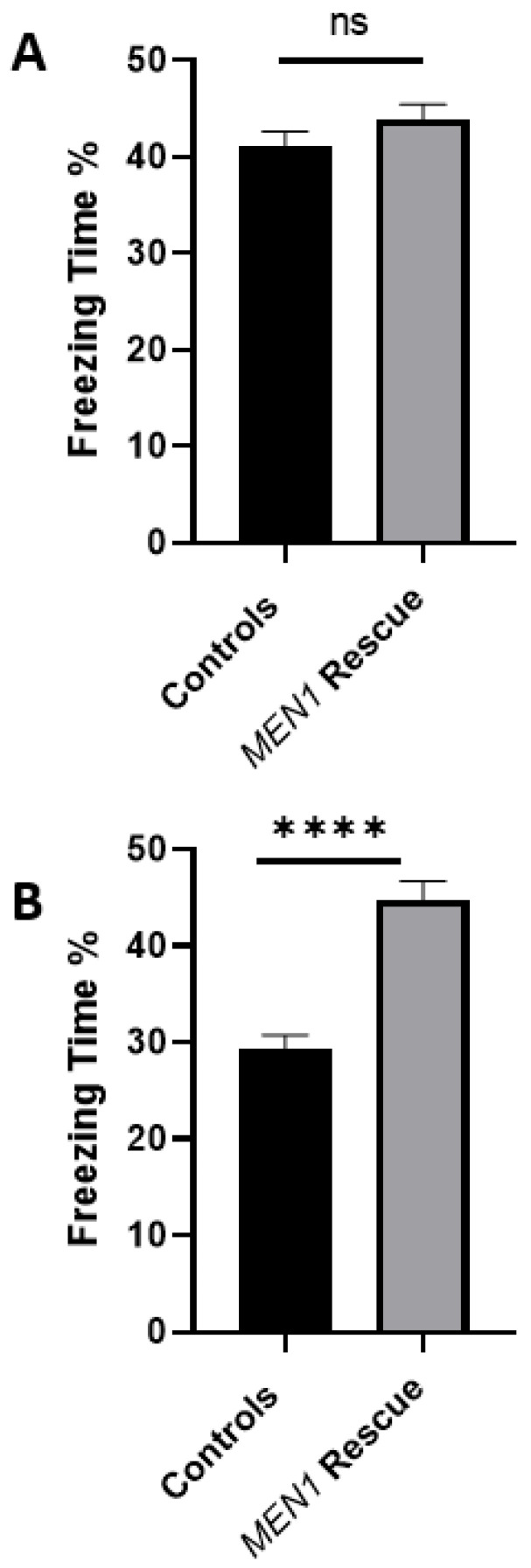
Contextual fear conditioning deficits reversed in the *MEN1* rescue mice. (**A**) For the contextual fear conditioning test, mice were trained and analyzed for freeze response. The scores of the freezing time (%) for the control mice and the *MEN1* rescue on day 1 were compared, and they show little difference; (**B**) *MEN1* rescue animals had a higher freezing time (%) score on Day 2 compared to the controls. Freezing response is the percentage of time spent freezing from the total time duration. Data represent the mean ± SEM. Here, n = 12 mice per group. Statistical significance (Mann–Whitney U test), **** *p* < 0.0001, ns *p* > 0.05.

After demonstrating that *MEN1* rescue is feasible, concomitant with the recovery of the menin expression, we then asked whether in vivo injection of *MEN1* virus in CKO animal could help rescue the behavioral deficit seen in those animals. Thus, we tested the hypothesis that *MEN1* reintroduction into *MEN1* CKO animals would rescue them from hippocampus-dependent fear conditioning. We first examined the health of the animals (feeding, locomotion, grooming, etc.) and then subjected them to a second round of stereotactic injections. We began with a total number of 16 CKO animals and 16 controls and, after testing these mice for the contextual fear conditioning task, sacrificed 4 CKO animals and 4 controls for the WB to confirm the CKO. The remaining animals were then split into 12 *MEN1* rescue animals and 12 controls. Specifically, the animals were anesthetized and immobilized on a platform. Both control and the experimental animals were subjected again to AAV protocol as before and allowed to recover from surgery for 28 days. The animals were continuously monitored for any side effects of surgery or the injections as before. We found that the mice remained healthy and exhibited normal locomotor, feeding, grooming, etc., behaviors. After 4 weeks, when we deemed *MEN1* expression to have occurred, these animals were tested again as above. Interestingly, while we found some age-related decline in the memory of the control animals, the AAV *MEN1*-injected animals retained memory to their original baseline levels. On day one of the contextual fear and conditioning test, the mean % time freezing of the *MEN1* rescue animals was 43.83%, and for the controls the mean % time freezing was 41.04%, showing no significant difference (Figure 11). On day two, the mean % time freezing score of the *MEN1* rescue animals was 44.67 %, and for the controls it was 29.31%, indicating a significant improvement in learning and memory, which was perturbed in the *MEN1* CKO model.

Taken together, the above data showed that the AAV capsids efficiently allowed for gene transfer to neurons of interest—albeit only in the hippocampal CA1 region—and resulted in the expression of menin. We next sought to determine if the deficit in the learning and memory seen in the CKO animals was reversed, concomitant with the recovery of menin protein expression. We observed that the rescued animals had a higher score compared to their control counterparts on day two of the task, which demonstrated that the mice were able to retrieve the fear conditioning memory in the same context. 

## 5. Discussion

Cholinergic transmission and plasticity play an important role in learning and memory. Perturbation of this pathway not only affects these processes, but also cognition, both of which are hallmarks of neurodegenerative diseases, such as AD. How cholinergic synaptic plasticity plays a role in the targeting of nAChRs to specific hippocampal synaptic sites remains, however, largely unknown. We previously demonstrated that a knockout of *MEN1* both in vitro and in vivo affects the localization of nAChRs [20,21]. Using the shRNA approach to knockdown menin expression, we have shown that upon menin deletion, the nAChR alpha 7 and alpha 5 were downregulated as determined by qPCR [21]. However, an ultimate test was needed to seek unequivocal evidence for the involvement of menin in learning and memory of freely behaving animals; this study has achieved just that. Moreover, we have further demonstrated that the reintroduction of MEN1 using a viral vector transfection in the live animals rescues the mice from learning and memory deficits induced by the knockout of the same gene.

Using different conditional knockout approaches, the current study has, on the one hand, identified the potential challenges which can arise when creating a gene knock down model in the CNS, while also underscoring the importance of alternative approaches. The tamoxifen-inducible approach, where the CamK2 Cre-Ert2 line was crossed with the *MEN1* floxed mice, did not yield a reasonable CKO model to work with, since no differences were observed at the protein level. Specifically, we discovered that when the CamK2 Cre-Ert2 line was crossed with the tdTomato reporter line, no difference in the expression of Cre recombinase was discernable, neither in the controls nor in the CKOs. Another shortcoming of this approach was that even though Camk2a-expressing neurons were present in the hippocampus, this also led to the deletion of menin from other regions, including the cortex and cerebellum. Since the aim of creating a CKO model was to test the role of menin specifically in the hippocampus, the loss of the gene from other regions was undesirable. Thus, a more direct approach was needed for specifically deleting *MEN1* from the hippocampus with complete spatiotemporal control. We then opted for a viral transduction approach and successfully employed it to delete menin exclusively in the hippocampal neurons with spatiotemporal control. Menin deletion was confirmed in both in vitro hippocampal cultures and in vivo—exclusively in the CA1 region of the hippocampus. Specifically, we demonstrated that there was a reduction in menin protein in the CKO model compared to the controls—both in the hippocampal culture and in the live animals.

Previous studies have used the Cre recombinase-loxP conditional knockout approach to study the tumor suppressor role of *MEN1* gene in the pituitary glands and pancreas [36]. A recent study showed the role of neuron-specific menin deletion on p35 and CdK5 pathways in the dysregulation and impairment of learning and memory [37]. This team created a conditional knockout model by crossing the *MEN1* floxed mice with CamKIIα-Cre line T29-1 transgenic mice that have the mouse Camk2a promoter for driving Cre recombinase expression in the forebrain, including the CA1 pyramidal cell layer of the hippocampus [37]. The downside of using this approach was that it did not allow for temporal control over menin deletion and the Cre recombinase led to menin deletion after birth. To overcome the lack of temporal control in inducing menin deletion, and the possibility of chromosomal effects, we used the approach of developing an inducible *MEN1* CKO model with the Camk2CreErt2 line. This was so that the leaky expression of Cre would not present itself as a problem. The tamoxifen-inducible model, CamK2CreErt2 crossed by *MEN1* floxed mice, also allowed for temporal control on the expression of Cre recombinase, which leads to *MEN1* deletion. Both the Camk2 Cre Ert 2 mice and the *MEN1* floxed mice were first genotyped to confirm the presence of Cre and the mutant menin. Since the tamoxifen protocol was already established, the next step was to quantify menin in the CKOs and controls after the mice were injected with tamoxifen. However, we were surprised to find equal fluorescence intensity in both the controls and CKOs in the WB; this approach was, therefore, deemed unsuitable.

Although the reason remains unclear, it has been reported elsewhere that lines, such as Nestin-Cre, GFAP-Cre, CaMKIIα-Cre, and Synapsin1-Cre, sometimes undergo unexpected germline recombination [38,39]. Other possible reasons could be genetic background effects, spontaneous mutations in the ERT2 or tamoxifen binding site, or unexpected genetic recombination [39]. Thus, we chose a more directed approach of deleting *MEN1* via virus transduction, which is discussed below.

An unexpected anomaly in the present study was the leaky expression of Cre in the hippocampus of the mice. The rationale for the ectopic expression could be that the titer of the virus was perhaps too high, resulting in its leakage into the vicinity of the hippocampus. Another possibility could be that the viral particles in the transduced neurons were jumping across the synapses. This explanation is, however, highly unlikely since it is not possible for AAVs to produce new viral particles [40]. The AAVs are specifically designed to infect only one node of the synaptic circuitry, and the concern of the virus infecting the neighboring neurons was, therefore, unwarranted [40]. 

There was a reduction in C-menin signal in the CKO compared to the controls both in vitro and in vivo. The reduction in menin in the CKO is indicative of reduced menin from neurons. The delivery of Cre to the hippocampus via the AAV approach is, thus, a viable approach both in vitro and in vivo. Generating the *MEN1* CKO model via injecting a neuron-specific Cre activated virus into the floxed *MEN1* to knock out *MEN1* function in the CA1 region allowed for precise spatial and temporal control. The *MEN1* was deleted in the CA1 region to draw a parallel between the 5XFAD model, where CA1 is one of the first regions where basal synaptic transmission and LTP start to deteriorate at around 6 months of age [41,42]. Furthermore, various subsets of the ventral CA1 neuron population play a role in the encoding and retrieval of contextual fear memories [43], which we deemed to be the evidence for a functional hippocampal neuron-specific knockout. The main goal of developing a CKO mouse model by specifically knocking down menin in the CA1 region was to determine its role in hippocampal-dependent learning and memory. A CKO model where menin expression is knocked down and validated with different approaches was needed before moving on to determine the role of the *MEN1* gene and menin protein in learning and memory. 

The data presented here demonstrate that *MEN1* CKO in a specific region of the CA1 had significant deficits in the hippocampal-dependent learning and memory task of contextual fear and conditioning. We had hypothesized that the CKO mice will exhibit deficits in hippocampal-dependent learning and memory, since *MEN1* gene deletion was shown previously to perturb the α7 nicotinic receptor expression [21]. Moreover, we had sought to also determine if *MEN1* reintroduction in the specific CA1 region, where this gene had initially been conditionally knocked out, could recover the observed behavioral deficit. Indeed, the data presented here are consistent with the hypothesis, thus, further establishing the role of *MEN1* and its encoded protein menin, in hippocampal-dependent, and neuronally contingent learning and memory. For the very first time, to our knowledge, a mouse model of menin CKO in the CA1 specific region was developed, and the effects of menin deletion from the CA1 on hippocampal-dependent learning and memory was demonstrated. The limitation of the experiment is that it remains unclear whether MEN1 overexpression alone causes an increase in freezing (in wildtype animals) and/or broad perturbations of motor behavior, which could influence performance on the fear conditioning test. Thus, there is a possibility that this may not be a true rescue experiment, since that control was not included and this possibility cannot be excluded based on the data reported.

Previously, menin has been shown to play a role in the regulation of nicotinic receptors at the postsynaptic site. Thus, if one were to delete menin as an ascribed regulator of synaptic clustering and recruitment of α7 nAChRs at the hippocampal synapses, then one could deduce its precise role in the targeting of these receptors. Furthermore, the CA1 sub-region undergoes disruption in the AD model; however, the underlying mechanisms as to why this perturbation occurs remain unknown [44]. Previously, CA1 region-specific alterations in the neuronal excitability have been reported in an AD mouse model [44]. Thus, to specifically highlight the importance of nicotinic machinery in the mouse model, the role of menin, which leads to perturbation of nicotinic receptors in the CA1 region, was essential.

A difference between the control and the experimental group was apparent for the contextual fear conditioning task. It has previously been shown that the aversive stimuli from hippocampal contextual representations during fear learning invoke the CA1 region [45]. Consistent with these observations, we found that after the deletion of menin from the CA1 region, the performance of the CKO mice in contextual and fear conditioning was impaired compared to the controls. This demonstrated that menin deletion led to the perturbation of circuitry in the CA1 region. Because the previous data from our lab had shown a direct correlation between menin and the α7 nAChRs, it, therefore, seems reasonable to conclude that the *MEN1* knockout-mediated loss of memory function had invoked these receptors. Future experiments will, however, be required to demonstrate this unequivocally, and may have to invoke either direct electrophysiology on brain slices, or optogenetic approaches, to determine the effects of menin deletion on the targeting of α7 nAChRs in freely behaving animals. 

The scores from the contextual fear conditioning task suggested that the CKO mice had their learning and memory impaired after *MEN1* deletion. Furthermore, our results corroborate with a recent study which demonstrated that *MEN1* CKO mice exhibited spatial learning and memory deficits [37]. This study reported that *MEN1* conditional knockout had deficits in the contextual fear and conditioning test, whereas they also did not find any difference in the cued conditional learning and memory. Our data expands on those findings, since the earlier CKO model employed in the Zhuang et al. study correlated the behavioral deficits in the CKO animals with a reduction in dendritic branching [37]. However, the approach to delete menin specifically from the CA1 region, and its reintroduction into the same region, shows the necessity and sufficiency of *MEN1* in learning and memory, which in turn is likely contingent upon synaptic clustering and recruitment of α7 nAChRs in the hippocampus. As anticipated, we have demonstrated that the *MEN1* CKO renders the animals dysfunctional vis-à-vis learning, memory, and cognitive function, which involves the disruption of cholinergic structures and function in the hippocampus. The reintroduction of *MEN1* in these CKO animals also restored the behavioral deficit concomitant with the recovery of menin function, which likely involved restoration of cholinergic transmission. Neurological disorders and diseases with underlying perturbation of the hippocampal nAChRs can be better understood by delineating the neural and cellular basis of how neural pathways and signaling mechanisms are altered. Thus, the role of menin in hippocampus-dependent learning and memory involving cholinergic circuits needs to be further studied. Another important takeaway from this study is that the genes that are ascribed some specific functions may in fact serve myriad other roles in organs different than those of their original target sites, thus, underscoring the importance of multifunctionality vis-à-vis genetic diversity.

## Data Availability

Data is available upon request.

## Supplementary Materials

The following supporting information can be downloaded at: https://www.mdpi.com/article/10.3390/cells11244019/s1, Figure S1: Cre expression in the cross between CamK2-CreErt2 and tdTomato mice.
