# Supplementary material for: Selective Menin Deletion in the Hippocampal CA1 Region Leads to Disruption of Contextual Memory in the MEN1 Conditional Knockout Mouse: Behavioral Restoration and Gain of Function following the Reintroduction of MEN1 Gene
Source: Cells. 2022 Dec 12;11(24):4019. doi: 10.3390/cells11244019 (PMC9776806; doi:10.3390/cells11244019)
Supplement: Supplementary file 1 [file cells-11-04019-s001.zip › cells-1901780-supplementary.pdf]

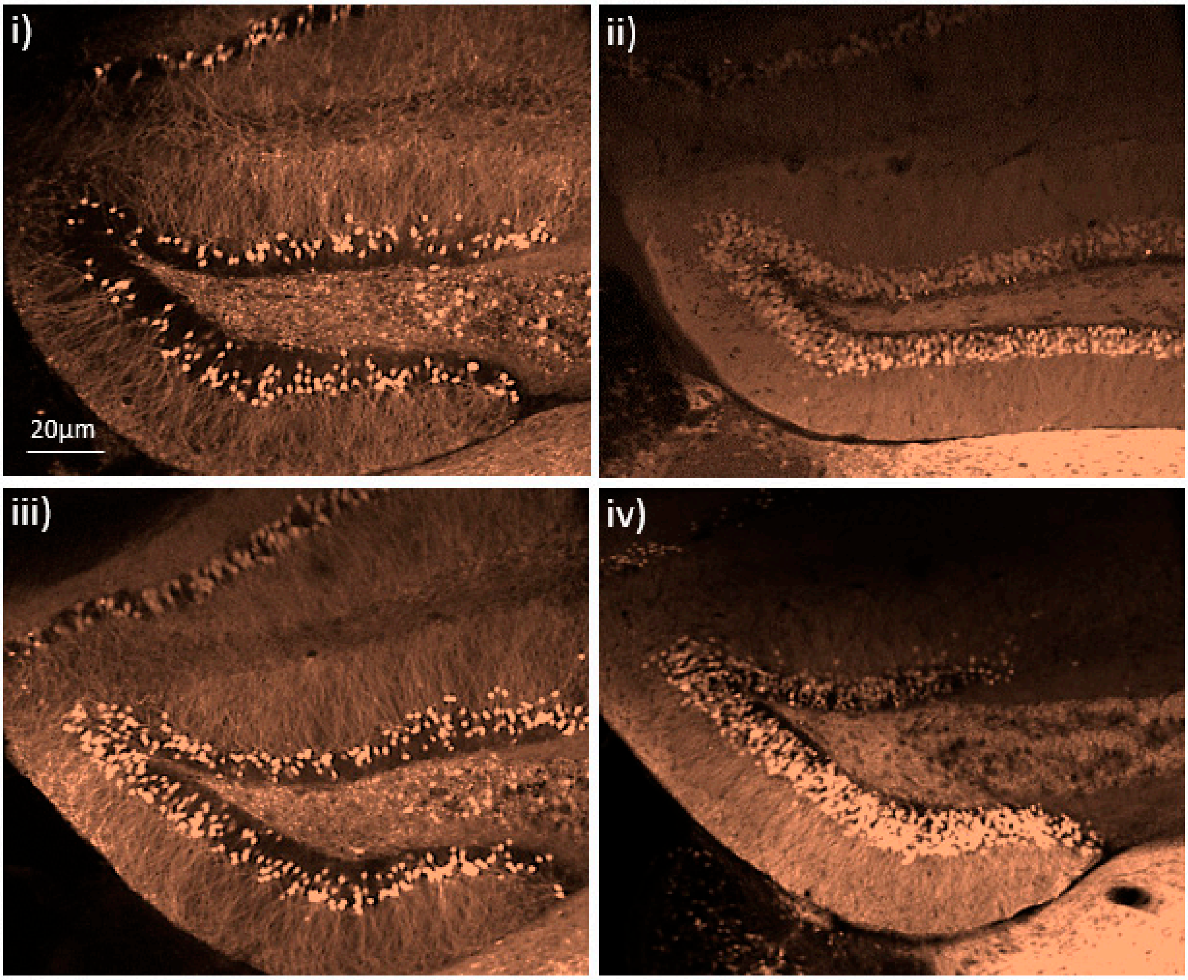

Supplementary Figure S1. Cre expression in the cross between CamK2-CreErt2 and tdTomato mice and tDTomato fluorescence showing Cre recombinase expression in both controls and CKO (i). Female controls injected with corn oil showing Cre expression (ii). Male controls injected with corn oil showing Cre expression (iii). Cre expressing females injected with tamoxifen showing Cre expression (iv). Cre expressing males injected with tamoxifen showing Cre expression. The Cre recombinase was leaky in both the corn oil and tamoxifen injected mice. Scale bars, 20μm.
